# Lymphangioleiomyomatosis diagnosed by transbronchial lung cryobiopsy in a patient with hemoptysis: a case report

**DOI:** 10.36416/1806-3756/e20260292

**Published:** 2026-11-09

**Authors:** Guilherme das Posses Bridi, Alexandre Franco Amaral, Luiza Escañuela Vaz, Gustavo Nogueira Schincariol Vicente, Evelise Lima, Ellen Caroline Toledo do Nascimento, Ronaldo Adib Kairalla, Bruno Guedes Baldi

**Affiliations:** 1. Divisão de Pneumologia, Instituto do Coração - InCor - Hospital das Clinicas -HCFMUSP - Faculdade de Medicina, Universidade de São Paulo, São Paulo (SP) Brasil.; 2. Departamento de Patologia, Faculdade de Medicina, Universidade de São Paulo, São Paulo (SP) Brasil.

## TO THE EDITOR:

Lymphangioleiomyomatosis (LAM) is a rare multisystem neoplastic disorder that predominantly affects women and is characterized by diffuse thin-walled pulmonary cysts caused by the proliferation of abnormal smooth muscle-like cells throughout the airways, vasculature, and lymphatic system.^1^ Although the diagnosis of LAM can often be established by the combination of characteristic HRCT findings and clinical manifestations consistent with the disorder-such as renal angiomyolipomas, chylous effusions, and tuberous sclerosis complex-with or without elevated serum VEGF-D levels, histopathological confirmation is necessary in some cases.^2^ In this context, transbronchial lung cryobiopsy has emerged as a less invasive alternative to surgical lung biopsy, providing larger and better-preserved tissue samples than does forceps biopsy and showing promising diagnostic performance in cystic lung diseases.^3^ ^,^ ^4^

Hemoptysis is an uncommon but recognized manifestation of LAM, potentially resulting from venous or capillary involvement due to infiltration of LAM cells into the pulmonary interstitium and vascular walls, leading to focal bleeding within the cystic lung parenchyma.^5^ This presentation usually raises initial suspicion of vasculitis, acute/chronic infections, and bronchiectasis, which makes the diagnosis of LAM particularly challenging in this context, especially in patients without a history of pneumothorax or known extrapulmonary features. Awareness of this less common clinical presentation in LAM is therefore essential to avoid diagnostic delay and to ensure prompt appropriate histological confirmation when required. Although occasional hemoptysis is reported in 10-15% of women with LAM,^6^ diffuse alveolar hemorrhage is rare, with only a few cases described in the literature to date.^5^ ^,^ ^7^ We report here the successful diagnosis of LAM obtained by transbronchial lung cryobiopsy in a patient presenting with intermittent hemoptysis.

A 40-year-old woman presented with a complaint of progressive dyspnea over the past several years. At this writing, her dyspnea is classified as grade 3 on the modified Medical Research Council dyspnea scale. The patient stated that she had experienced no previous episodes of pneumothorax or pleural effusion but reported intermittent hemoptysis and occasional blood-streaked sputum. Her past medical history was notable for uterine leiomyomatosis with dysfunctional uterine bleeding, for which she had used progesterone-only therapy on an irregular basis. She also had a history of systemic hypertension and depressive mood disorder. She had had only one pregnancy, more than ten years prior, and had conceived without the need for hormone therapy; she underwent surgery for an endometrioma in the subsequent year. She was a never-smoker and reported no other features of autoimmune diseases. Lung auscultation was unremarkable, and her SpO_2_ was 99% on room air. She tested negative for autoantibodies, including antinuclear antibodies. The VEGF-D levels were not measured. Pulmonary function test results were normal. Chest CT demonstrated multiple bilateral diffuse thin-walled cysts, together with widespread ground-glass opacities throughout the lung parenchyma (Figure 1). Abdominal CT showed no significant abnormalities. There were no clinical or CT manifestations suggestive of the presence of tuberous sclerosis complex. Transbronchial lung cryobiopsy was performed in the lateral and posterior segments of the left lower lobe, yielding a total of six tissue samples. Histopathological examination revealed the presence of LAM cells with positive immunohistochemical staining for smooth muscle actin, HMB-45, and β-catenin, along with prominent intra-alveolar hemorrhage (Figure 2). No procedure-related complications occurred.

**Figure 1:**
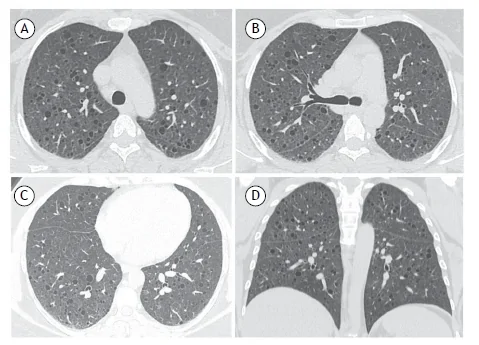
HRCT showing multiple bilateral, diffuse, thin-walled pulmonary cysts accompanied by widespread ground-glass opacities.

**Figure 2:**
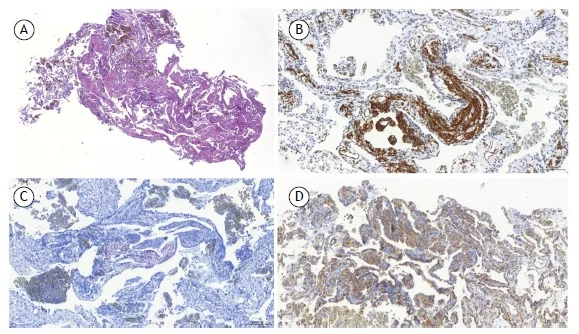
Histopathological and immunohistochemical features of lymphangioleiomyomatosis on transbronchial lung cryobiopsy. (A) Hematoxylin and eosin staining demonstrating prominent intra-alveolar hemorrhage. (B) Smooth-muscle actin positivity in spindle-shaped cells within the lesion. (C) HMB-45 positivity. (D) Nuclear and cytoplasmic β-catenin expression in LAM cells.

This case highlights an unusual presentation of LAM with hemoptysis, expanding the spectrum of pulmonary bleeding manifestations in diffuse cystic lung diseases. Although hemoptysis has been sporadically reported in patients with LAM, exuberant alveolar hemorrhage on histopathology is distinctly uncommon and may reflect pulmonary vascular involvement by LAM cells.^5^ In our patient, the absence of autoimmune markers, together with the typical cystic pattern on chest CT and the definitive immunohistochemical profile, confirmed the diagnosis of LAM. This report also underscores the value of transbronchial lung cryobiopsy as a less invasive, safe, and effective diagnostic modality for selected patients with diffuse cystic lung disease, particularly when the clinical-radiological presentation is inconclusive.

Albeit rare, LAM is a neoplastic cystic lung disease that predominantly affects women of reproductive age and may occur sporadically or in combination with tuberous sclerosis complex. The source of LAM cells is not fully established, although recent evidence strongly suggests a uterine origin.^8^ The most common clinical manifestations include progressive exertional dyspnea, recurrent pneumothorax, and chylous pleural effusions, whereas extrapulmonary associations such as renal angiomyolipomas and lymphangioleiomyomas (abdominal and pelvic) may provide important diagnostic clues.^1^ ^,^ ^9^

Current guidelines support establishing the diagnosis of LAM on the basis of the combination of a characteristic HRCT pattern of diffuse, bilateral, thin-walled cysts and confirmatory clinical, extrapulmonary, or serological features, particularly serum VEGF-D levels ≥ 800 pg/mL.^1^ ^,^ ^9^ When tissue confirmation is required in diffuse cystic lung disease, the choice of biopsy technique is particularly relevant. Although conventional transbronchial forceps biopsy may provide diagnostic material in LAM, especially at centers with expertise and when multiple samples are obtained, its effectiveness is often limited by small fragment size, crush artifact, and insufficient preservation of the alveolar-interstitial architecture, potentially reducing the sensitivity of immunohistochemical staining for markers such as HMB-45 and smooth-muscle actin.^3^ ^,^ ^10^ Surgical lung biopsy remains the historical reference standard, particularly in atypical cystic patterns; however, its use is constrained by higher morbidity, postoperative pain, prolonged hospitalization, and a non-negligible risk of pneumothorax or persistent air leak in patients with cystic pulmonary lesions.^3^ ^,^ ^10^

In this scenario, transbronchial lung cryobiopsy has emerged as an attractive strategy, combining the less invasive nature of bronchoscopy with the ability to obtain substantially larger and better-preserved specimens than forceps biopsy. This is particularly advantageous in diffuse cystic lung diseases, in which diagnostic structures may be focal and located in the cyst wall, perivascular interstitium, or adjacent alveolated lung parenchyma. A retrospective study comparing cryobiopsy and forceps biopsy in patients with suspected LAM found similar diagnostic yields (66.7% and 66.0%, respectively), while cryobiopsy provided larger specimens with better preservation of tissue architecture, supporting its role as a valuable diagnostic alternative in selected cases.^3^ Although concerns remain regarding pneumothorax and bleeding, careful fluoroscopic guidance, selection of less destroyed parenchymal areas adjacent to cystic lesions, and the use of bronchial blockers have made the procedure increasingly safe at experienced centers.^3^ ^,^ ^4^ ^,^ ^10^ The safety of transbronchial lung cryobiopsy in the management of diffuse cystic lung diseases is supported by the absence of complications presented by the patient in this report.

Once a diagnosis of LAM has been established, sirolimus is currently the first-line therapy for patients with abnormal or declining lung function and may also be beneficial in chylous effusions and other extrapulmonary complications.^1^ ^,^ ^9^ Although hemoptysis may occur in LAM, it is considerably less common than is pneumothorax or progressive dyspnea, and marked intra-alveolar hemorrhage on histopathology is distinctly unusual. Pulmonary vascular involvement by LAM cells may help explain the occurrence of hemoptysis, typically in patients with a high LAM cell burden, possibly triggered by circumstances associated with increased sympathetic stimulation, such as sexual activity or vigorous exercise.^11^ The combination of diffuse thin-walled cysts and ground-glass opacities in this context broadens the differential diagnosis to include diffuse alveolar hemorrhage related to capillaritis (secondary to an autoimmune process), infection, smoking-related interstitial disorders, and other diffuse cystic lung diseases. Our case supports the growing role of cryobiopsy as an effective diagnostic alternative to confirm the diagnosis and better clarify less common presentations of LAM.
